# Does “Lying Flat” Lead to Greater Life Satisfaction? Evidence from Empirical Research

**DOI:** 10.3390/bs15081067

**Published:** 2025-08-06

**Authors:** Huanhua Lu, Jinli Wang, Feng Kong

**Affiliations:** 1 School of Marxism, China University of Geosciences (Beijing), Beijing 100083, China; 2 School of Psychology, Shaanxi Normal University, Xi’an 710062, China

**Keywords:** “lying flat”, life satisfaction, temporal directionality, cross-lagged analyses

## Abstract

At present, faced with intense competitive stress, some young people have adopted a lifestyle of “lying flat”—a passive attitude characterized by giving up efforts and goals—in order to relieve pressure and increase life satisfaction. However, the question of whether “lying flat” can really increase life satisfaction is still unclear. Our study attempted to investigate the relation between “lying flat” and life satisfaction. We combined cross-sectional (Study 1) and longitudinal research (Study 2) to investigate the relation between “lying flat” and life satisfaction. Study 1 showed that “lying flat” was significantly negatively associated with life satisfaction, and the cross-lagged analyses in Study 2 found that “lying flat” significantly negatively predicted life satisfaction one month later, but life satisfaction did not significantly predict “lying flat” one month later. The present study first revealed a temporal directionality between the “lying flat” and life satisfaction. This suggests that “lying flat”, which serves as a temporary relief mechanism in the face of overwhelming pressure, may come at the cost of long-term psychological functioning.

## 1. Introduction

The “lying flat” phenomenon is particularly prevalent among younger generations in China, including millennials and Generation Z, who are grappling with the pressures of navigating an increasingly competitive job market and societal expectations (Ou, 2023). Literally, “lying flat” refers to a person lying flat on his or her back, with the whole body relaxed, making no movement and responding to nothing (Y. Chen & Zhang, 2023). In this state, the individual seems to be indifferent to the outside world and does not respond to any stimuli. This attitude of “inaction”, “no effort”, is the sociological derivation of the word “lying flat” (Ou, 2023). Many scholars have discussed the characteristics of “lying flat” and agreed that individuals truly “lying flat” showed the following characteristics: no goals, no effort, and no action (Y. Chen & Cao, 2021; C. Deng, 2022; Hu & Wang, 2023; Lin & Gao, 2021; Ling & Wang, 2023; C. Ma & Wang, 2022; Qin & Dai, 2022; Wei & Wang, 2023). From a cultural perspective, the emergence of the “lying flat” phenomenon in China reflects a tension between traditional Confucian values—emphasizing diligence, perseverance, and societal contribution—and the modern pressures of hyper-competition, economic uncertainty, and social stratification (Confucius, 2014). As a localized response to these evolving dynamics, “lying flat” may represent not just a psychological reaction, but a cultural adaptation to increasingly perceived futility in achieving success through conventional effort. While “lying flat” has gained particular visibility in China, similar sentiments are observable globally. For instance, in Western societies, movements like “quiet quitting” or the rise of NEETs (Not in Employment, Education, or Training) reflect young people’s disillusionment with traditional success narratives in the context of unstable job markets and escalating social pressures (Eurofound, 2012). Comparing “lying flat” with global phenomena can provide a broader framework for understanding youth disengagement in different socio-economic systems.

Life satisfaction refers to a cognitive evaluation of one’s life as a whole (Diener et al., 2009; Pavot & Diener, 2008). It involves an individual’s judgment or assessment of the overall quality of their life based on their own criteria (Diener et al., 2009; Pavot & Diener, 2008). This evaluation often takes into account various domains of life such as work, income, relationships, and health, and reflects a person’s contentment with their life circumstances (Diener et al., 2009; Pavot & Diener, 2008). “Lying flat” is an alternative lifestyle chosen by some young people who are dissatisfied with the status quo of life, so can “lying flat” improve an individual’s life satisfaction? Some young people claimed in online forums that “lying flat” was a way of life independently chosen by individuals in the face of pressure, reflecting the subjectivity of human beings, and that “lying flat” can alleviate pressure and anxiety and make individuals happier, with higher levels of life satisfaction (Cao, 2020; Feng, 2021; Luo, 2021; Z. Ma, 2021; Sun et al., 2021; X. Wang, 2018, 2021, 2022; Yang, 2021). In this sense, “lying flat” might represent a rational and adaptive response to social conditions perceived as unjust or excessively demanding. When structural barriers hinder upward mobility, “lying flat” may serve as a form of psychological self-preservation, minimizing stress and protecting mental health in the short term, which may temporarily increase an individual’s life satisfaction. However, most theorists argue that truly “lying flat” (i.e., giving up the pursuit of goals, making no effort, and taking no action) may cause an individual to lose their enthusiasm and motivation for life, and it will affect the individual’s personal growth and career development, which will in turn decrease their life satisfaction (Y. Chen & Zhang, 2023; Q. Liu, 2023; Song & Qin, 2023; J. Wang, 2021; Xiang, 2021). The relation between “lying flat” and life satisfaction is controversial, but no empirical studies have explored it yet. The purpose of our study is to combine cross-sectional and longitudinal studies to examine the relationship between the two.

Although no studies have yet explored the relation of “lying flat” and life satisfaction, some theories can give us some insights. Self-Determination Theory posits that individuals are motivated to pursue activities that satisfy three basic psychological needs: autonomy, competence, and relatedness (Chirkov et al., 2003; Deci & Ryan, 2000, 2008; Deci et al., 2001; Ryan & Deci, 2000). When individuals engage in hard work and when they feel competent in their abilities to accomplish tasks, they are more likely to experience a sense of fulfillment and satisfaction, contributing to overall life satisfaction (Diener et al., 2003; Ferreira et al., 2020; T. Liu et al., 2019; Morales-García et al., 2024; Žnidaršič & Marič, 2021). And individuals engaging in “lying flat” do not work hard or do not make an effect, making it difficult to gain a sense of competence, which is not conducive to life satisfaction. Goal-Setting Theory posits that setting specific, challenging goals leads to increase motivation and performance (Locke & Latham, 2006; Tubbs, 1986). When individuals set ambitious goals and work diligently toward achieving them, they experience a sense of accomplishment and satisfaction upon goal attainment, which contributes to overall life satisfaction (Schunk, 1990; Sheldon & Elliot, 1999; W. Wang et al., 2017). And individuals engaging in “lying flat” do not have clear goals and are not willing to work hard, which makes it difficult to gain a sense of accomplishment and is not conducive to increasing life satisfaction. Positive Psychology emphasizes the importance of cultivating strengths, virtues, and positive experiences to enhance well-being and life satisfaction (Fredrickson, 2001; M. Seligman et al., 2005). From this perspective, working hard can be viewed as a means of self-improvement, personal growth, and leading to greater overall life satisfaction. And individuals who engage in “lying flat” do not have clear achievement goals, do not work hard, and do not make progress, thus losing opportunities for self-improvement and personal growth, which is not conducive to increasing life satisfaction (Ryff, 1989; Sheldon & Houser-Marko, 2001; Waterman, 1993; Weigold et al., 2020). Therefore, the above theories give us new insights, indicating that the higher the degree of “lying flat”, the worse the life satisfaction.

Additionally, previous studies have confirmed that life satisfaction was influenced by factors such as income, achievement goals, work engagement, and work hours (Ding et al., 2021; Ferreira et al., 2020; FitzRoy & Nolan, 2022; Hsu et al., 2019; T. Liu et al., 2019; Morales-García et al., 2024; Shao, 2022; Sheldon & Elliot, 1999; W. Wang et al., 2017; Žnidaršič & Marič, 2021). Income that can meet basic needs (such as food, shelter, and healthcare) was found to be positively correlated with life satisfaction (Ding et al., 2021; FitzRoy & Nolan, 2022). Progress toward meaningful goals that can boost individuals’ sense of competence was also demonstrated to be associated with greater levels of life satisfaction (Sheldon & Elliot, 1999; W. Wang et al., 2017). A positive association between work engagement and life satisfaction was also found (Ferreira et al., 2020; T. Liu et al., 2019; Morales-García et al., 2024; Žnidaršič & Marič, 2021). That is, when individuals are fully engaged in their work, experiencing a sense of purpose, involvement, and enthusiasm, it often spills over into other areas of their life, contributing to higher levels of overall life satisfaction (Ferreira et al., 2020; T. Liu et al., 2019; Morales-García et al., 2024; Žnidaršič & Marič, 2021). Research suggested that too much free time reduced life satisfaction, and there was an optimal number of working hours conducive to life satisfaction (Hsu et al., 2019; Shao, 2022). Individuals who engage in “lying flat” (have no goals, do not work hard, and do not make progress), will have a lower income, which cannot meet basic needs; at the same time, it is difficult to experience a sense of purpose, sense of competence, and sense of fulfillment, and this makes individuals prone to falling into ruminative thinking, all of which will lead to a decline in life satisfaction (Deci & Ryan, 2000; FitzRoy & Nolan, 2022; Huppert & So, 2013; Nolen-Hoeksema et al., 2008; Ryff, 1989; Sheldon & Niemiec, 2006). Therefore, it is reasonable to put forward the following hypothesis: the higher the degree of “lying flat”, the worse the life satisfaction.

In this study, we sought to explore the relation between “lying flat” and life satisfaction. We combined a cross-sectional (Study 1) and longitudinal study (Study 2) to explore the relation of “lying flat” and life satisfaction. We proposed that “lying flat” would be negatively associated with life satisfaction and that there would be temporal directionality, meaning that “lying flat” would negatively predict life satisfaction over time. Each study (Study 1 and Study 2) utilized independent samples.

## 2. Study 1

### 2.1. Method

#### 2.1.1. Participants

Participants were selected through a stratified multistage sampling design. (1) All higher education institutions in Beijing were stratified into key universities and ordinary colleges; (2) one institution per stratum was randomly selected using computer-generated random numbers; (3) within each institution, 500 full-time undergraduates were systematically sampled from official registries; and (4) the counselor in charge of students invited the selected students to fill in the anonymous survey link via campus email or WeChat. Each participant received set remuneration after completing the survey. Participants did not have any prior personal contact with the researchers. In the Chinese higher education system, “key universities” refer to institutions included in the national “Double First-Class”, “Project 985”, or “Project 211” initiatives, which receive prioritized government funding and are typically more prestigious. In contrast, “ordinary colleges” refer to universities outside these projects, with comparatively fewer academic and financial resources. A total of 480 questionnaires were collected from the key university, with a recovery rate of 96%, while 492 questionnaires were collected from the ordinary college, with a recovery rate of 98.4%. After removing 12 invalid questionnaires with incomplete or regular answers, we finally obtained 960 valid questionnaires, of which 470 were from the key university and 490 were from the ordinary college. The average age of the participants was 20.30 ± 2.142 (range 18–28), and other basic information is shown in Table 1.

#### 2.1.2. Measures

“Lying flat”: “Lying flat” was assessed using the Lying Flat Tendency Scale (LFTS) developed by Lu et al. (2023). This scale includes six items such as “I do not have any goals and pursuits for life and study.” Participants respond using a four-point scale (1—“quite inconsistently”, 2—”relatively inconsistently”, 3—”relatively consistently”, and 4—”quite consistently”). Higher overall scores on this questionnaire indicate a greater degree of “lying flat.” If the participant chooses less than or equal to 2 for all 6 items, it means that he/she is comparatively unlikely to engage in “lying flat”; if the participant chooses greater than or equal to 3 for all 6 items, it meant that he/she is more likely to engage in “lying flat”. Accordingly, we can divide out several score bands according to the participants’ total scores on the scale; participants with total scores between 6 and 12 are less likely to engage in “lying flat”, and those with total scores between 18 and 24 are more likely to engage in “lying flat”. While we used a cutoff score of 18 to descriptively identify participants with high “lying flat” tendencies, all inferential analyses treated the total score on the LFTS as a continuous variable. In this study, the Cronbach’s α coefficient for the LFTS was 0.849.

Life satisfaction: The life satisfaction was measured by the life satisfaction dimension on the Index of Well-being (IWB) (Campbell et al., 1976), which is widely used to assess life satisfaction (Cummins et al., 2003; Diener et al., 1985; Helliwell et al., 2014; Proctor et al., 2009). The dimension consists of 2 items (e.g., I am very dissatisfied with my life in general) and is scored on a 7-point scale. In this study, the items were reverse scored, and the higher the total scores, the higher the life satisfaction. In this study, Cronbach’s α coefficient for this dimension was 0.800.

In addition, this study measured the participants’ basic information such as gender, age, university/college attended, and subjective socioeconomic status (SSES).

#### 2.1.3. Statistical Analysis

First, we used descriptive statistics to analyze the current situation of the youth’s “lying flat” phenomenon, and then we used correlation analysis and hierarchical regression to examine the association between the degree of “lying flat” and life satisfaction. Hierarchical regression was structured with two distinct models. Model 1 was formulated with life satisfaction as the dependent variable, incorporating participants’ basic demographic information (gender, age, SSES, and university/college attended) as independent variables. Subsequently, Model 2 expanded upon the initial model by integrating “lying flat” as an additional predictor. Ultimately, the impact of “lying flat” on life satisfaction was assessed by comparing the incremental variance accounted for by each model. All statistics were performed utilizing SPSS 26.0.

### 2.2. Results

#### 2.2.1. Current Situation of the Youth’s “Lying Flat” Phenomenon

**Figure 1:**
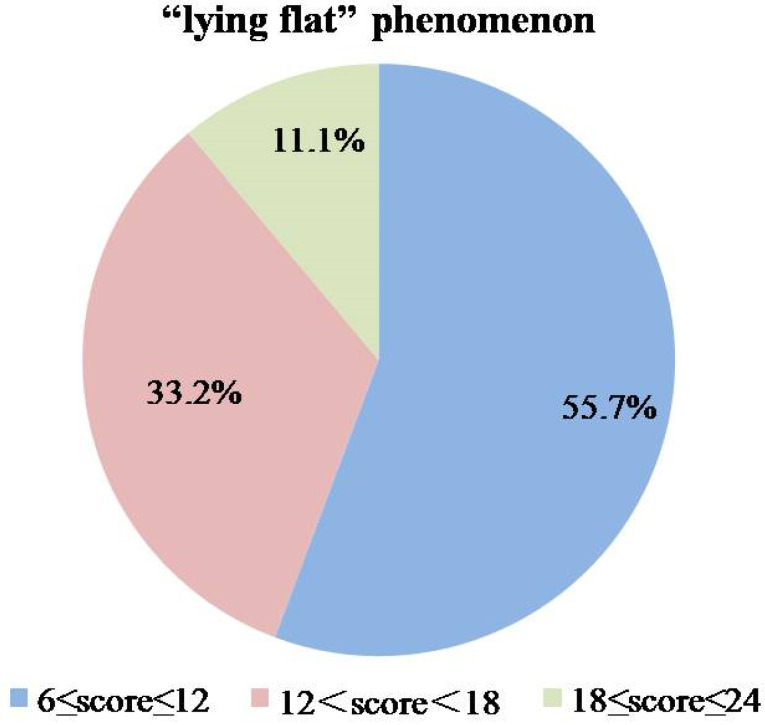
The proportion of subjects in each score band of the LFTS.

Descriptive statistics showed that 11.1% of youth were more likely to engage in “lying flat”, with total scores of the LFTS between 18 and 24. Figure 1 shows the proportion of participants in each score band.

**Figure 2:**
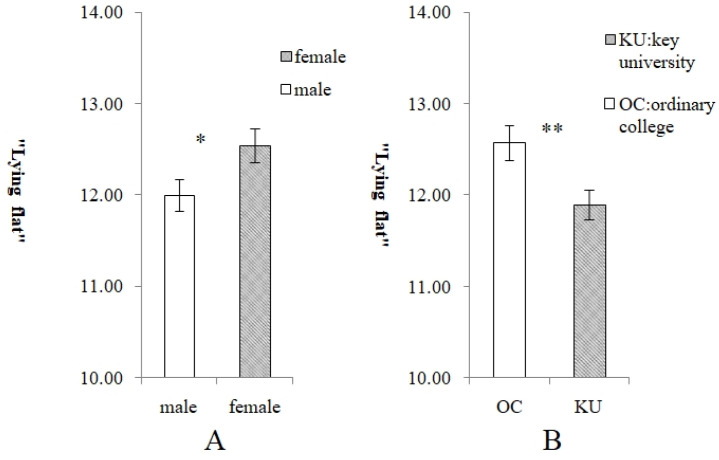
Gender difference and institutional differences concerning “lying flat”. (**A**) Gender difference concerning “lying flat”. (**B**) Institutional differences concerning “lying flat”. ** *p* < 0.01; * *p* < 0.05.

The results of the independent samples *t*-test showed that the degree of “lying flat” of male participants (*M* = 12.00, *SD* = 3.989) was significantly lower than that of female participants (*M* = 12.55, *SD* = 3.856) (*t* = −2.152, *p* = 0.032) (Figure 2A); the degree of “lying flat” of youth from the ordinary college (*M* = 12.58, *SD* = 4.267) was significantly higher than that of youth from the key university (*M* = 11.90, *SD* = 3.533) (*t* = −2.693, *p* = 0.007) (Figure 2B). One-way ANOVA showed that there was no significant difference in “lying flat” among individuals with different SSESs (high/medium/low) (*F* = 2.342, *p* = 0.097). Additionally, the correlation analysis showed that there was no significant correlation between age and “lying flat” (*r*= −0.028, *p* = 0.388).

#### 2.2.2. Common-Method Bias Test

To assess potential common-method bias (CMB), we conducted Harman’s single-factor test, a widely used preliminary diagnostic technique in behavioral research (Podsakoff et al., 2003; Fuller et al., 2016). Although this approach has known limitations—such as its inability to detect all sources of method variance—it remains a commonly accepted first step for evaluating CMB, particularly in cross-sectional survey designs (W. G. Deng et al., 2018). Thus, we used it as an initial check for potential bias. The result showed that there was no serious CMB in this study, with the first factor having an explanatory rate of 33.090% (<40% criterion).

Importantly, the present study also included a longitudinal component (Study 2), in which key variables were measured at different time points. This temporal separation serves as a design-based remedy to reduce the likelihood of CMB and strengthens the inference of causal direction. Longitudinal designs are widely regarded as an effective means of mitigating method bias through procedural separation (Podsakoff et al., 2003).

#### 2.2.3. The Relationship Between “Lying Flat” and Life Satisfaction

Correlation analysis revealed a significant negative correlation between the degree of “lying flat” and life satisfaction (*r* = −0.447, *p* < 0.001). Then, a hierarchical regression analysis (Table 2) demonstrated that Model 1 accounted for 5.6% of the variance in life satisfaction, whereas Model 2 significantly increased the explanatory power to 25.2%. This enhancement in the Model’s variance explained was largely attributable to the inclusion of “lying flat”. That is say, after controlling for the effects of participants’ basic demographic information, “lying flat” emerged as a significant negative predictor of life satisfaction (β = −0.446, *p* < 0.001), underscoring its robust influence on the predicted variable.

## 3. Study 2

### 3.1. Participants

We recruited 120 participants from a university for a longitudinal follow-up study. Excluding 11 loss participants, we obtained a total of 109 valid responses 65 males and 44 females, with a mean age of 19.09 ± 0.80 (range 18–22). G*power 3.1 was used to perform sample size estimation, which showed that a minimum of 80 participants were needed to obtain 80% statistical power, with α = 0.05 and *r* = 0.3. Therefore, the number of participants complied with the requirement in this study.

#### Experimental Procedures

We recruited 120 participants for this study. At the first time point (T1), all 120 participants completed the questionnaire, including “lying flat”, life satisfaction, and participants’ basic information. After one month, we repeated the measurements at a second time point (T2) by re-administering the questionnaire to these participants. Since 11 participants did not complete the questionnaire, we ultimately obtained 109 valid responses.

### 3.2. Measures

“Lying flat”: the LFTS (Lu et al., 2023), the same as in Study 1, was used to assess “lying flat” (at T1: Cronbach’s α = 0.823, at T2: Cronbach’s α = 0.876).

Life satisfaction: the life satisfaction dimension in the Index of Well-being (IWB) (Campbell et al., 1976), the same as in Study 1, was used to assess life satisfaction (at T1: Cronbach’s α = 0.896, at T2: Cronbach’s α = 0.913).

In addition, this study also measured the participants’ basic information such as gender, age, and SSESs.

### 3.3. Statistical Analysis

**Figure 3:**
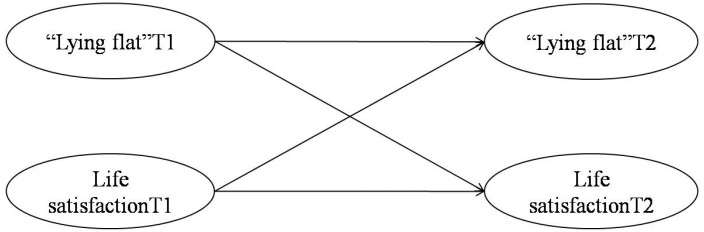
Cross-lagged model of “lying flat” and life satisfaction.

The data were meticulously analyzed using SPSS 26.0 and AMOS 22. Initially, both descriptive statistics and a common method bias test were performed, followed by a correlation analysis, all executed within the SPSS environment. Subsequently, the longitudinal measurement equivalence of the experimental variables (the LFTS and life satisfaction dimension of the IWB) was assessed using AMOS 22. The final phase of the analysis entailed a latent variable cross-lag panel analysis, also conducted with AMOS 22. This sophisticated analytical technique involved the construction of a structural equation model (SEM) (Figure 3) to delineate the dynamic relation between “lying flat” and life satisfaction. The SEM incorporated autoregressive paths for the primary variables, reflecting the temporal stability of each construct. Additionally, cross-lagged paths were specified to capture the reciprocal influences from “lying flat” at T1 to life satisfaction at T2 and, conversely, from life satisfaction at T1 to “lying flat” at T2. These cross-lagged paths are instrumental in discerning the directionality of effects over time.

### 3.4. Results

#### 3.4.1. Descriptive Statistical Analysis

Table 3 presents the mean, standard deviation, kurtosis, and skewness of “lying flat” and life satisfaction at both time points. These results indicated that the data for “lying flat” and life satisfaction were generally normally distributed at both time points in this study.

#### 3.4.2. CMB Test

CMB test with Harman’s one-way method (Podsakoff et al., 2003) showed that there was no serious CMB in this study, with the first factor having an explanatory rate of 33.078% (<40% criterion) at T1, and the first factor having an explanatory rate of 37.677% (<40% criterion) at T2.

#### 3.4.3. Correlation Analysis Between “Lying Flat” and Life Satisfaction

Correlation analyses conducted at each respective time point revealed a significant negative correlation between “lying flat” and life satisfaction (At T1, *r* = −0.267, *p* = 0.005, and at T2, *r* = −0.310, *p* = 0.001, Table 4). Furthermore, “lying flat” exhibited a significant positive autocorrelation between T1 and T2 (*r* = 0.669, *p* < 0.001, Table 4), indicating strong stability in “lying flat” across the two time points. Similarly, life satisfaction also exhibited a significant positive autocorrelation between T1 and T2 (*r* = 0.476, *p* < 0.001, Table 4), suggesting that individuals’ life satisfaction was relatively stable across the two time points. These autocorrelations underscored the temporal reliability of both “lying flat” and life satisfaction.

#### 3.4.4. Longitudinal Measurement Equivalence Tests for Measured Variables

Measurement equivalence tests were performed across time for the LFTS and life satisfaction dimension of the IWB. Four steps were included: (1) a morphological equivalence test, (2) a weak equivalence test, (3) a strong equivalence test, and (4) a strict equivalence test (F. F. Chen, 2007). The results showed that the fitting indices of all four models were in the acceptable range (Table 5), and the change in both CFI and RMSEA was less than 0.01 when comparing M2 and M1 and comparing M3 and M2 (Table 5), but the change in CFI was more than 0.01 when comparing M4 and M3 (Table 5). As suggested by F. F. Chen’s (2007), when the change in both CFI and RMSEA was less than 0.01, the measurement equivalence test was passed. Thus, our findings suggested that the LFTS and life satisfaction dimension of the IWB passed the strong equivalence test but not the strict equivalence test.

#### 3.4.5. Cross-Lagged Analysis of “Lying Flat” and Life Satisfaction

Following the significant negative correlations identified through correlation analysis between “lying flat” and life satisfaction at both two time points, a cross-lagged panel model was employed to investigate the potential temporal directionality between the two. This analysis was performed using Amos 22, and the result showed that the model had acceptable fit indices (*χ*^2^*/df* = 1.737, CFI = 0.928, TLI = 0.909, RMSEA = 0.083, SRMR = 0.0648). The autoregressive paths within the model indicated significant temporal stability for both “lying flat” (β = 0.818, *p* < 0.001, Table 6) and life satisfaction (β = 0.382, *p* < 0.001, Table 6), reinforcing the consistency of these constructs over time. Notably, the cross-lagged paths revealed a significant negative predictive effect of “lying flat” at T1 on life satisfaction at T2 (β = −0.404, *p* < 0.001, Table 6). Conversely, life satisfaction at T1 did not exert a significant predictive effect on “lying flat” at T2 (β = 0.117, *p* = 0.151, Table 6). Subsequent cross-lagged analyses, controlling for participants’ basic demographic information (gender, age, and SSES), confirmed the robust negative predictive effect of “lying flat” at T1 on life satisfaction at T2 (β = −0.414, *p* < 0.001). Collectively, these findings suggested that “lying flat” may precede and potentially predict variations in life satisfaction, offering valuable insights into the dynamics between these two constructs.

## 4. Discussion

In this study, we revealed the relation of “lying flat” and life satisfaction for the first time. The cross-sectional study found that there was a significant negative correlation between the degree of “lying flat” and life satisfaction, and the longitudinal study found that “lying flat” negatively predicted life satisfaction one month later; i.e., there was a temporal directionality between the two.

Our study found that 11.1% of young participants were more likely to engage in “lying flat”, and, based on OECD statistics for 2024, the proportion of “NEET” youth in most OECD member countries was above 10% (OECD, 2024), which indicates that the number of young people who are “lying flat” is not small around the world. Furthermore, the implications of “lying flat” should also be understood within broader socio-cultural frameworks. In Chinese society, deeply rooted in Confucianism, values such as industriousness, perseverance, and the pursuit of upward mobility have long been emphasized (Confucius, 2014). However, with the rise of hyper-competition, economic inequality, and limited social mobility, young individuals increasingly perceive traditional ideals as unattainable. Consequently, “lying flat” may function as a means of symbolic resistance to entrenched social expectations.

Our study also found that the degree of “lying flat” was significantly higher in the youth from the ordinary college than from the key university. In China, individuals who were admitted to the key university needed to perform better academically, so they needed to expend more effort and had more resilience in the face of difficulties, compared with those who were admitted to the ordinary college. Therefore, in the face of the ever-involving social environment, the youth from the key university were less likely to choose to engage in “lying flat” than those from the ordinary college.

Our study also found that the degree of “lying flat” of the female participants was significantly higher than that of the male participants. While this may be partly rooted in traditional gender socialization, in which men are expected to be ambitious and resilient, and women are encouraged to be cautious and risk-averse (Ebrey, 1993; Huang, 1990; Johnson, 1983; Mann, 1997; Dai, 2010), it is also important to consider the evolving and often conflicting expectations placed on modern women. In contemporary Chinese society, women are increasingly expected to excel academically and professionally while also fulfilling traditional family roles. This dual burden may lead to heightened stress, fatigue, and ultimately disengagement. Structural barriers such as gender discrimination in the workplace, wage inequality, and pressures related to marriage and childbearing further undermine women’s perceived control over their life trajectories and future outcomes. As a result, some women may adopt “lying flat” as a form of psychological self-protection or coping in response to the limited and conflicting options available to them.

More importantly, we found temporal directionality between “lying flat” and life satisfaction; that is, “lying flat” significantly negatively predicted life satisfaction. Individuals engaging in more “lying flat” behaviors (giving up pursuing their goals, not working hard, and not making progress) may have difficulty not only in obtaining opportunities for high pay but also in obtaining opportunities for personal growth and realizing achievement goals, all of which have a negative impact on life satisfaction (Deci & Ryan, 2000; FitzRoy & Nolan, 2022; Ryff, 1989; Sheldon & Houser-Marko, 2001; Waterman, 1993; Weigold et al., 2020). On the other hand, this may also be related to the fact that all the participants in this study were Chinese, and that in Chinese culture, it has always been emphasized that “Heaven rewards the hard-working” and that happiness is achieved through struggle (Confucius, 2014), with those who do not work hard and do not make progress behaving contrarily to the traditional culture, making it difficult to gain social acceptance, which is not conducive to increasing life satisfaction. This study was the first to use empirical research to prove that the higher the degree of “lying flat”, the lower the life satisfaction. It extends the existing literature on the determinants of subjective well-being by highlighting the negative consequences of avoidance-oriented motivational patterns—an area often underexplored in contrast to the emphasis on positive psychological resources such as autonomy, purpose, and engagement.

It is important to note that although our findings demonstrate a significant negative relationship between “lying flat” and life satisfaction, this association may not be entirely attributable to the behavioral stance itself. Some participants may adopt “lying flat” not as a free choice but in response to perceived structural constraints—such as limited opportunities or excessively high societal expectations—which in turn may reduce both motivation and life satisfaction. This psychological process aligns more closely with the notion of “learned helplessness”, in which individuals feel incapable of effecting change in their environment and thus respond with withdrawal and resignation (M. E. P. Seligman, 1975). In this sense, “lying flat” may reflect broader issues of systemic pressure and psychological strain rather than a simple motivational deficit. Future research should explore the underlying motivations for “lying flat” and distinguish between active “lying flat” and passive “lying flat”, potentially through experimental manipulations or structural equation modeling to account for such confounding factors.

### 4.1. Implications

This study was the first to use empirical research to find the temporal directionality between “lying flat” and life satisfaction; that is, “lying flat” significantly negatively predicted life satisfaction. This deepens our theoretical understanding of “lying flat” as a psychological coping strategy. Rather than mere behavioral withdrawal, “lying flat” may serve as a temporary relief mechanism in the face of overwhelming pressure yet may come at the cost of long-term psychological functioning. This insight contributes to ongoing discussions about adaptive versus maladaptive disengagement.

In addition to its theoretical contributions, this study also offers valuable practical implications. In educational settings, understanding the psychological roots of the “lying flat” attitude could help develop programs that promote intrinsic motivation, goal clarity, and resilience among students. Schools and universities might consider creating more autonomy-supportive environments and fostering a sense of purpose beyond conventional academic achievement. In workplace contexts, organizations could address early-career disengagement by designing jobs that offer meaningful tasks, flexible goal-setting, and opportunities for personal development. Emphasizing employee well-being and psychological needs might prevent youth burnout and turnover. At the policy level, these results call for reflection on systemic sources of youth dissatisfaction—such as housing unaffordability, hyper-competition, and job insecurity. Public initiatives that alleviate structural pressures and provide pathways for self-realization may help reduce the appeal of “lying flat” as a coping mechanism.

### 4.2. Limitations

This study also has some limitations. First, the exclusive use of a Chinese university student sample limits the generalizability of the findings. These participants were at a particular developmental stage and were exposed to specific societal pressures—such as academic competition, employment uncertainty, and shifting identity expectations—which might have intensified the observed association between “lying flat” and life satisfaction. Future research should examine more diverse samples across different age groups, educational backgrounds, occupational settings, and cultural contexts to assess the robustness and universality of the findings.

Second, the operationalization of the “lying flat” construct in this study was relatively narrow, focusing primarily on motivational withdrawal and behavioral disengagement. This may have caused more nuanced or contextually adaptive forms of “lying flat” to be overlooked. For example, some individuals may adopt “lying flat” as a reflective or strategic coping mechanism in response to overwhelming societal demands. Such forms of disengagement may not be inherently maladaptive. Future work should develop more refined and context-sensitive measurement tools that can differentiate between various types and functions of “lying flat”.

Third, although we conceptualize “lying flat” as a novel sociocultural construct, it may have a conceptual overlap with existing psychological constructs such as amotivation, learned helplessness, or apathy. These constructs similarly capture motivational collapse or disengagement from goals and expectations. Future research should consider comparing and integrating these frameworks to clarify whether “lying flat” represents a culturally specific manifestation of these phenomena or constitutes an independent motivational stance.

Finally, the longitudinal design in Study 2 involved only a one-month interval. While this design allowed for the identification of temporal directionality, longer follow-up periods are needed to evaluate the stability and long-term implications of the observed effects.

## 5. Conclusions

This study found a temporal directionality between “lying flat” and life satisfaction for the first time; that is, “lying flat” significantly negatively predicted life satisfaction. This not only contributes to understanding the psychological consequences of “lying flat” among Chinese youth but also opens the door for cross-cultural comparisons of youth disengagement. Future research should explore how similar expressions of withdrawal or disengagement manifest across different cultures, and how societal values, labor market conditions, and generational expectations interact to shape such attitudes. Understanding these nuances is essential for developing culturally appropriate strategies to support youth well-being in a rapidly changing world.

## Figures and Tables

**Table 1:** Descriptive statistical analysis of participants’ basic information.

Variables		*n*	%
Gender	Male	532	55.4
	Female	428	44.6
University/college attended	Key university	470	49.0
	General college	490	51.0
Family economic status	Low	140	14.5
	Medium	710	74
	High	110	11.5

**Table 2:** Hierarchical regression analysis of “lying flat” and life satisfaction.

Variables	Life Satisfaction									
	Model 1					Model 2				
	*B*	*SE*	*β*	*t*	*p*	*B*	*SE*	*β*	*t*	*p*
gender	0.156	0.171	0.029	0.914	0.361	0.306	0.153	0.056	2.004	0.045
age	0.287	0.050	0.228	5.765	<0.001	0.310	0.044	0.246	7.003	<0.001
university attended	−0.012	0.213	−0.002	−0.055	0.956	0.241	0.190	0.045	1.267	0.206
family economic status	0.094	0.046	0.065	2.058	0.040	0.084	0.041	0.058	2.071	0.039
“lying flat”						−0.306	0.019	−0.446	−15.863	<0.001
*F*	14.081					64.539				
Δ*F*	14.081					251.635				
*R^2^*	0.056					0.252				
Δ*R^2^*	0.056					0.197				

**Table 3:** Descriptive statistical analysis of main variables.

Variables	*M ± SD*	*Max*	*Min*	*Skewness*	*Kurtosis*
“Lying flat” T1	11.53 ± 3.436	24	6	0.429	0.568
Life satisfaction T1	10.45 ± 2.061	14	3	−0.385	0.648
“Lying flat” T2	12.99 ± 3.794	24	6	0.019	−0.181
Life satisfaction T2	9.94 ± 2.123	14	4	−0.027	−0.249

**Table 4:** Correlation analysis between “lying flat” and life satisfaction.

	1	2	3	4
1 “Lying flat” T1	—			
2 “Lying flat” T2	0.669 ***	—		
3 life satisfaction T1	−0.267 **	−0.132	—	
4 life satisfaction T2	−0.454 ***	−0.310 ***	0.476 ***	—

*** *p* < 0.001; ** *p* < 0.01, two-tailed test.

**Table 5:** Longitudinal measurement equivalence tests for measured variables.

Model	*χ* ^2^	*df*	*CFI*	*TLI*	*SRMR*	*RMSEA*	*Model Comparison*	Δ*CFI*	Δ*RMSEA*
M1	62.342	28	0.959	0.917	0.0577	0.075			
M2	69.932	34	0.957	0.929	0.0632	0.07	M2-M1	−0.002	−0.005
M3	72.121	37	0.958	0.936	0.0796	0.066	M3-M2	0.001	−0.004
M4	90.555	45	0.945	0.932	0.0804	0.068	M4-M3	−0.013	0.002

Notes: M1 = morphological equivalence model; M2 = weak equivalence model; M3 = strong equivalence model; M4 = strict equivalence model.

**Table 6:** Cross-lagged analysis of “lying flat” and life satisfaction.

Autoregressive Pathway	*β*	*p*	Cross-Lagged Path	*β*	*p*
“Lying flat” T1 → “Lying flat” T2	0.818	<0.001	“Lying flat” T1→Life satisfaction T2	−0.404	<0.001
Life satisfaction T1 → Life satisfaction T2	0.382	<0.001	Life satisfaction T1→ “Lying flat” T2	0.117	0.151

## Data Availability

Data can be available on https://osf.io/rq8dv/?view_only=9f6af0e65b864ea7b6680e9a8935dbe3 (accessed on 10 May 2025).

## List of Abbreviations

LFTS	Lying flat tendency scale
IWB	Index of well-being
SSES	Subjective socioeconomic status
CMB	Common method bias
T1	First time point
T2	Second time point
SEM	Structural equation model

## References

[B1-behavsci-15-01067] Campbell A., Converse P. E., Rodgers W. L. (1976). The quality of American life: Perceptions, evaluations, and satisfactions.

[B2-behavsci-15-01067] Cao J. (2020). The defense of “Destigmatization” of “Mourning” culture in the perspective of emotional consumption. Radio & TV Journal.

[B3-behavsci-15-01067] Chen F. F. (2007). Sensitivity of goodness of fit indexes to lack of measurement invariance. Structural Equation Modeling.

[B4-behavsci-15-01067] Chen Y., Cao Y. (2021). “Lying Flat”: Emergence, formation mechanisms and social consequences. Fujian Tribune.

[B5-behavsci-15-01067] Chen Y., Zhang L. (2023). A study of the Internet buzzword “lying flat”. Sinogram Culture.

[B6-behavsci-15-01067] Chirkov V., Ryan R. M., Kim Y., Kaplan U. (2003). Differentiating autonomy from individualism and independence: A self-determination theory perspective on internalization of cultural orientations and well-being. Journal of Personality and Social Psychology.

[B7-behavsci-15-01067] Confucius, Confucius (2014). Counsels of the Great Yu. Shang shu.

[B8-behavsci-15-01067] Cummins R. A., Eckersley R., Pallant J., van Vugt J., Misajon R. (2003). Developing a national index of subjective wellbeing: The australian unity wellbeing index. Social Indicators Research.

[B9-behavsci-15-01067] Dai S., Dai S. (2010). Nei Ze. The book of rites. (Warring states to western Han period).

[B10-behavsci-15-01067] Deci E. L., Ryan R. M. (2000). The “what” and “why” of goal pursuits: Human needs and the self-determination of behavior. Psychological Inquiry.

[B11-behavsci-15-01067] Deci E. L., Ryan R. M. (2008). Facilitating optimal motivation and psychological well-being across life’s domains. Canadian Psychology/Psychologie Canadienne.

[B12-behavsci-15-01067] Deci E. L., Ryan R. M., Gagne M., Leone D. R., Usunov J., Kornazheva B. P. (2001). Need satisfaction, motivation, and well-being in the work organizations of a former eastern bloc country: A cross-cultural study of self-determination. Personality and Social Psychology Bulletin.

[B13-behavsci-15-01067] Deng C. (2022). Commentary on the “lying-flatters”: Identity, cultural symptoms and ideological guidance. Studies in Ideological Education.

[B14-behavsci-15-01067] Deng W. G., Li X. Y., Chen B., Luo K., Zeng X. Y. (2018). The current status of common method bias testing in domestic psychological literature. Journal of Jiangxi Normal University (Natural Sciences).

[B15-behavsci-15-01067] Diener E., Emmons R. A., Larsen R. J., Griffin S. (1985). The satisfaction with life scale. The Journal of Personality Assessment.

[B16-behavsci-15-01067] Diener E., Oishi S., Lucas R. E. (2003). Personality, culture, and subjective well-being: Emotional and cognitive evaluations of life. Annual Review of Psychology.

[B17-behavsci-15-01067] Diener E., Oishi S., Lucas R. E. (2009). Subjective well-being: The science of happiness and life satisfaction.

[B18-behavsci-15-01067] Ding J., Salinas-Jiménez J., Salinas-Jiménez M. d. M. (2021). The impact of income inequality on subjective well-being: The case of China. Journal of Happiness Studies.

[B19-behavsci-15-01067] Ebrey P. B. (1993). The inner quarters: Marriage and the lives of Chinese women in the sung period.

[B20-behavsci-15-01067] Eurofound (2012). NEETs—Young people not in employment, education or training: Characteristics, costs and policy responses in Europe.

[B21-behavsci-15-01067] Feng H. (2021). “Laying flat”: Whose justice? What kind of justification?.

[B22-behavsci-15-01067] Ferreira P., Gabriel C., Faria S., Rodrigues P., Sousa Pereira M. (2020). What if employees brought their life to work? The relation of life satisfaction and work engagement. Sustainability.

[B23-behavsci-15-01067] FitzRoy F. R., Nolan M. A. (2022). Income status and life satisfaction. Journal of Happiness Studies.

[B24-behavsci-15-01067] Fredrickson B. L. (2001). The role of positive emotions in positive psychology: The broaden-and-build theory of positive emotions. American Psychologist.

[B25-behavsci-15-01067] Fuller C. M., Simmering M. J., Atinc G., Atinc Y., Babin B. J. (2016). Common methods variance detection in business research. Journal of Business Research.

[B26-behavsci-15-01067] Helliwell J., Huang H., Wang S. (2014). Social capital and well-being in times of crisis. Journal of Happiness Studies.

[B27-behavsci-15-01067] Hsu Y. Y., Bai C. H., Yang C. M., Huang Y. C., Lin T. T., Lin C. H. (2019). Long hours’ effects on work-life balance and satisfaction. BioMed Research International.

[B28-behavsci-15-01067] Hu Z., Wang Y. (2023). Modelling and coping strategies for the phenomenon of “laying flat” among youths. Journal of Hubei University of Economics (Humanities and Social Sciences).

[B29-behavsci-15-01067] Huang P. C. C. (1990). The peasant family and rural development in the Yangzi delta, 1350–1988.

[B30-behavsci-15-01067] Huppert F. A., So T. T. (2013). Flourishing across Europe: Application of a new conceptual framework for defining well-being. Social Indicators Research.

[B31-behavsci-15-01067] Johnson K. A. (1983). Women, the family, and peasant revolution in China.

[B32-behavsci-15-01067] Lin L., Gao Y. (2021). “Lying-Flat Youth”: A structural dilemma explanation. China Youth Study.

[B33-behavsci-15-01067] Ling X., Wang Y. (2023). “Involution”, “Buddhism”, and “Lying Flat”: Conceptual evolution, boundary layers, and corrective strategies—An interpretation based on the perspective of philosophy of culture. Journal of Xinjiang Normal University (Edition of Philosophy and Social Sciences).

[B34-behavsci-15-01067] Liu Q. (2023). Study on the dissolution of the phenomenon of youth “Laying Flat” on the mainstream ideology and strategies to cope with it. Journal of Guangxi Youth Leaders College.

[B35-behavsci-15-01067] Liu T., Zeng X., Chen M., Lan T. (2019). The harder you work, the higher your satisfaction with life? The influence of police work engagement on life satisfaction: A moderated mediation model. Frontiers in Psychology.

[B36-behavsci-15-01067] Locke E. A., Latham G. P. (2006). New directions in goal-setting theory. Current Directions in Psychological Science.

[B37-behavsci-15-01067] Lu H., Hou J., Huang A., Wang J., Kong F. (2023). Development and validation of the “Lying Flat” tendency scale for the youth. Behavioral Sciences.

[B38-behavsci-15-01067] Luo H. (2021). Lying flat is righteous.

[B39-behavsci-15-01067] Ma C., Wang Y. (2022). The group characteristics, epochal factors and coping strategies of “Lying Flat-ism”. Ideological & Theoretical Education.

[B40-behavsci-15-01067] Ma Z. (2021). “Lying Flat”: An alternative posture to resist deep alienation. Exploration and Free Views.

[B41-behavsci-15-01067] Mann S. (1997). Precious records: Women in China’s long eighteenth century.

[B42-behavsci-15-01067] Morales-García W. C., Vallejos M., Sairitupa-Sanchez L. Z., Morales-García S. B., Rivera-Lozada O., Morales-García M. (2024). Depression, professional self-efficacy, and job performance as predictors of life satisfaction: The mediating role of work engagement in nurses. Frontiers in Public Health.

[B43-behavsci-15-01067] Nolen-Hoeksema S., Wisco B. E., Lyubomirsky S. (2008). Rethinking rumination. Perspectives on Psychological Science.

[B44-behavsci-15-01067] OECD (2024). Youth not in employment, education or training (NEET) (indicator).

[B45-behavsci-15-01067] Ou X. (2023). The social mentality, behavioral patterns and guidance of “Lying-flat” youths. Journal of Chongqing University of Posts and Telecommunications (Social Science Edition).

[B46-behavsci-15-01067] Pavot W., Diener E. (2008). The satisfaction with life scale and the emerging construct of life satisfaction. The Journal of Positive Psychology.

[B47-behavsci-15-01067] Podsakoff P. M., MacKenzie S. B., Lee J.-Y., Podsakoff N. P. (2003). Common method biases in behavioral research: A critical review of the literature and recommended remedies. Journal of Applied Psychology.

[B48-behavsci-15-01067] Proctor C. L., Linley P. A., Maltby J. (2009). Youth life satisfaction: A review of the literature. Journal of Happiness Studies.

[B49-behavsci-15-01067] Qin X., Dai Y. (2022). “Involution”, “Buddhist” to “Lying Flat”—Cultivating the spirit of youth struggle from the changes of social mentality. China Youth Study.

[B50-behavsci-15-01067] Ryan R. M., Deci E. L. (2000). Self-determination theory and the facilitation of intrinsic motivation, social development, and well-being. American Psychologist.

[B51-behavsci-15-01067] Ryff C. D. (1989). Happiness is everything, or is it? Explorations on the meaning of psychological well-being. Journal of Personality and Social Psychology.

[B52-behavsci-15-01067] Schunk D. H. (1990). Goal setting and self-efficacy during self-regulated learning. Educational Psychologist.

[B54-behavsci-15-01067] Seligman M., Steen T., Park N., Peterson C. (2005). Positive psychology progress: Empirical validation of interventions. The American Psychologist.

[B53-behavsci-15-01067] Seligman M. E. P. (1975). Helplessness: On depression, development, and death.

[B55-behavsci-15-01067] Shao Q. (2022). Does less working time improve life satisfaction? Evidence from European social survey. Health Economics Review.

[B56-behavsci-15-01067] Sheldon K. M., Elliot A. J. (1999). Goal striving, need satisfaction, and longitudinal well-being: The self-concordance model. Journal of Personality and Social Psychology.

[B58-behavsci-15-01067] Sheldon K. M., Houser-Marko L. (2001). Self-concordance, goal attainment, and the pursuit of happiness: Can there be an upward spiral?. Journal of Personality and Social Psychology.

[B57-behavsci-15-01067] Sheldon K. M., Niemiec C. P. (2006). It’s not just the amount that counts: Balanced need satisfaction also affects well-being. Journal of Personality and Social Psychology.

[B59-behavsci-15-01067] Song Y., Qin M. (2023). Characterization analysis, theoretical causes and guiding paths of youth “pendulum culture”. Youth and Adolescent Studies.

[B60-behavsci-15-01067] Sun S., Li W., Lu X., Liu H. (2021). Elephant breakouts and young man’s lying flat: How to find a new order in a shifting system. Youth Research.

[B61-behavsci-15-01067] Tubbs M. E. (1986). Goal setting: A meta-analytic examination of the empirical evidence. Journal of Applied Psychology.

[B62-behavsci-15-01067] Wang J. (2021). Self-deconstructing layaboutism—A critique of “layabout justice theory”. Exploration and Free Views.

[B63-behavsci-15-01067] Wang W., Li J., Sun G., Cheng Z., Zhang X.-A. (2017). Achievement goals and life satisfaction: The mediating role of perception of successful agency and the moderating role of emotion reappraisal. Psicologia: Reflexão e Crítica.

[B64-behavsci-15-01067] Wang X. (2018). Buddhism is a negative good. Exploration and Free Views.

[B65-behavsci-15-01067] Wang X. (2021). Analysis of the phenomenon of synthetic syndromes lying flat. Exploration and Free Views.

[B66-behavsci-15-01067] Wang X. (2022). Rational criticism of lying—Flat doctrine. Journal of Guangzhou University (Social Science Edition).

[B67-behavsci-15-01067] Waterman A. (1993). Two conceptions of happiness: Contrasts of personal expressiveness (eudaimonia) and hedonic enjoyment. Journal of Personality and Social Psychology.

[B68-behavsci-15-01067] Wei D., Wang Y. (2023). “Involution” versus “lying flat”, discourse representation and behavioral performance of contemporary youth: A study based on rootedness theory. Youth and Adolescent Studies.

[B69-behavsci-15-01067] Weigold I., Weigold A., Russell E., Wolfe G., Prowell J., Martin-Wagar C. (2020). Personal growth initiative and mental health: A meta-analysis. Journal of Counseling and Development: JCD.

[B70-behavsci-15-01067] Xiang Y. (2021). Disenchanting and reconstructing: The social roots and cultural reflection of “lying flat culture”. Social Sciences in Xinjiang.

[B71-behavsci-15-01067] Yang Y. (2021). “Lying flat”: The emotional expression of youth subculture. Social Sciences Weekly.

[B72-behavsci-15-01067] Žnidaršič J., Marič M. (2021). Relationships between work-family balance, job satisfaction, life satisfaction and work engagement among higher education lecturers. Organizacija.

